# Molecular Investigations of Protein Aggregation in the Pathogenesis of Amyotrophic Lateral Sclerosis

**DOI:** 10.3390/ijms24010704

**Published:** 2022-12-31

**Authors:** Elisa Duranti, Chiara Villa

**Affiliations:** School of Medicine and Surgery, University of Milano-Bicocca, 20900 Monza, Italy

**Keywords:** amyotrophic lateral sclerosis, aggregation, neurodegenerative diseases

## Abstract

Amyotrophic lateral sclerosis (ALS) is a devastating progressive neurodegenerative disorder characterized by selective loss of lower and upper motor neurons (MNs) in the brain and spinal cord, resulting in paralysis and eventually death due to respiratory insufficiency. Although the fundamental physiological mechanisms underlying ALS are not completely understood, the key neuropathological hallmarks of ALS pathology are the aggregation and accumulation of ubiquitinated protein inclusions within the cytoplasm of degenerating MNs. Herein, we discuss recent insights into the molecular mechanisms that lead to the accumulation of protein aggregates in ALS. This will contribute to a better understanding of the pathophysiology of the disease and may open novel avenues for the development of therapeutic strategies.

## 1. Introduction

Amyotrophic lateral sclerosis (ALS) is an idiopathic disease characterized by progressive skeletal muscle paralysis caused by degeneration of the lower and/or upper motor neurons (MNs), resulting in severe disability [1]. ALS is a fatal disease that results in death caused by respiratory failure 3–5 years after diagnosis [2]. The rate of disease progression varies between individuals and can be affected by the site of onset, but it is usually rapid [3]. Comparative analyses of various European data confirm that ALS is estimated to have an annual incidence rate of 2–3/100,000 people, with a prevalence of 8–10/100,000 people in the West. This disease can affect people of any age, but onset under 20 years is extremely rare, and incidence rises to a peak between the ages of 65 and 75, after which it falls again. Moreover, Caucasians and males are slightly more likely to develop ALS than females, with a 1.2 times higher risk [3]. Unfortunately, available drugs cannot stop the disease but can only provide temporary relief and extend patients’ median survival by a few months [4].

Approximately 85–90% of ALS cases are sporadic (sALS), indicating a complex interaction between genetic and environmental risk factors. The remaining 10–15% of ALS cases are generally dominantly inherited and are thus classified as familial (fALS) [5]. Although over 30 ALS-related genes have been identified, the majority of fALS forms are caused by mutations in genes encoding superoxide dismutase 1 (*SOD1*), transactive response (TAR)-DNA binding protein 43 (*TARDBP*), fused in sarcoma (*FUS*), and chromosome 9 open reading frame 72 (*C9ORF72*) [6,7]. Proteins encoded by these genes are involved in a variety of biochemical processes and cellular pathways, such as autophagy, protein quality control, RNA metabolism, mitochondria, and ATP homeostasis. Interestingly, sALS and fALS are clinically indistinguishable [2].

The exact etiology of the disease is unknown; however, clinical evaluations have revealed the involvement of multiple cellular functions in patients’ MNs, abnormal increase in excitatory muscle tone, accumulation of misfolded protein, impaired axonal transport, and high calcium metabolism. Numerous studies have reported that the death of MNs is not due to a single event, but is caused by a combination of various mechanisms [8], including oxidative stress, mitochondrial dysfunction, cytotoxicity, the formation of protein aggregates, and changes in RNA metabolism [9,10,11]. The accumulation of insoluble proteins at the MN level is a key feature of ALS pathology. Most of these cytoplasmic inclusions are ubiquitinated [12] and contain primarily either TDP-43 [13,14], SOD1 [15], or FUS [16,17] proteins.

As these abnormal aggregates are toxic for cells and are responsible for neurodegeneration, this review discusses the molecular mechanisms underlying impaired protein homeostasis, also called proteostasis, in the pathogenesis of ALS.

## 2. Proteinopathies

Neurodegenerative diseases are usually age-related and characterized by a slow and progressive loss of different nerve functions. Among them, the most common are Alzheimer’s disease (AD), Parkinson’s disease (PD), Huntington’s disease (HD), and ALS [18]. The majority of neurodegenerative diseases, with the exception of HD, can have a genetic or sporadic etiology, and familial forms typically manifest earlier with a more severe phenotype. In most cases the onset occurs in the fourth or fifth decade of life. Neurodegenerative disorders share common risk factors that include aging, oxidative stress, environmental stress, and protein malfunction. All of these affect cellular proteostasis, the process that maintains the proteome in the proper concentration (balancing protein production and degradation), in the correct folding (chaperones), and in the right place at the right time (trafficking). Two main cellular degradation systems prevent the formation of impaired proteins: the ubiquitin-proteasome system (UPS), which degrades functional as well as dysfunctional proteins, and the autophagy–lysosomal system (ALP) responsible for the degradation of whole organelles, large aggregates of proteins or macromolecules, and single proteins [19]. From a pathophysiological point of view, different pathologies are distinguished by the synthesis of aberrant proteins that can undergo conformational changes and acquire a toxic function or block their original biological activity [20]. Based on this feature, such diseases are classified as proteinopathies.

Although the proteins that play physiological roles in a healthy brain have monomeric structures, pathological conditions can cause them to undergo conformational changes that favor their association and result in the formation of oligomers, which can eventually aggregate into higher-order structures. These aggregates typically precipitate in different brain areas. Under these conditions, proteins might either acquire harmful qualities or cease their physiological function. Neurodegenerative disorders and proteinopathies are characterized by protein accumulation, both wild-type (wt) and mutant, with the altered conformation favoring aggregation [21]. These accumulations, which include TDP-43, FUS, SOD1, tau, and α-synuclein (α-syn), can develop intracellularly and in the surrounding area, as in the case of amyloid β (Aβ), and can determine the pathogenic forms of disease.

A single form of protein aggregation may cause certain proteinopathies, while others may be caused by multiple types. Proteinopathies are frequently mixed disorders, making their diagnosis and treatment challenging. Furthermore, they frequently share clinical features with other diseases. An example of this is the presence in AD brains of neurofibrillary tangles comprising tau aggregates and Aβ plaques, along with the accumulations of α-syn typical of Lewy body disease and PD. This characteristic is also found in ALS, which exhibits aggregates of proteins including TDP-43/FUS/SOD1, α-syn, tau, or Aβ [22,23,24].

Toxic accumulation may emerge from any of these phenomena, including for example enhanced translation of a particular mRNA, transcriptional activation, or a lower rate of protein degradation due to impairment of the proteasomal pathway [21,25].

## 3. Pathological Protein Aggregation Involved in ALS

**Figure 1:**
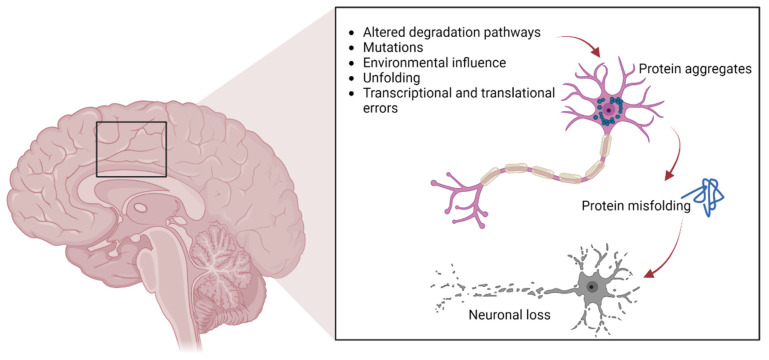
The pathways involved in protein aggregation in neuronal cells, leading to neuronal death typically found in neurodegenerative diseases.

As previously mentioned, protein aggregates are a pathological feature of several neurodegenerative diseases, including extracellular plaques of Aβ and intracellular neurofibrillary aggregates of tau protein in AD, or Lewy bodies in PD [26]. Pathological protein aggregates are also a feature of ALS, and occur in the form of ubiquitinated inclusions in neurons and glia (Figure 1).

Such inclusions contain different proteins, some of which may have an intrinsic tendency to aggregate following genetic mutations (SOD1, TDP-43, FUS), whereas others may simply be trapped in the aggregates [13,14,15,16,17,27]. In particular, cysteine-mediated aggregates of mutant SOD1 (mutSOD1) have been observed in ALS MNs, with the wt form of SOD1 also present, thus demonstrating the strong affinity and co-aggregation of the two forms of the protein [28]. It is widely believed that the toxic functions of mutSOD1 and other proteins typical of ALS are related to their tendency to aggregate. This hypothesis is supported by the presence of intracellular cytoplasmic inclusions, as well as mitochondrial inclusions rich in SOD1 identified in cell models and in animal spinal MNs, as well as in MNs of patients with ALS [27]. 

Numerous experimental theories have been proposed to explain how the aggregation of mutant proteins contributes to cellular toxicity in ALS patients. These suggestions have included the ability to sequester proteins necessary for the normal functioning of the MN [29], the ability to reduce the activity of the proteasome, which is essential for correct protein turnover, and the ability to inhibit the correct functioning of specific cellular organelles, such as mitochondria, by internal or external aggregation [30]. Cell degeneration depends on the sensitivity of MNs to the aggregation of proteins in the mutant form, supported by the observation thatTDP-43 and FUS also aggregate in patient tissues and ALS models in the same manner as SOD1 [31,32]. Indeed, anatomopathological observations of tissues derived from ALS patients show that these two proteins aggregate as cytoplasmic inclusions (positive for ubiquitin, but negative for SOD1) and that the mutations that affect them seem to increase the degree of aggregation [33]. Furthermore, it has been shown that the aggregation of FUS and TDP-43 is based on a conserved low-complexity domain (LCD), also called a prion-like domain (PrLD) [34], and that such aggregates can also sequester wt proteins and in their native form [10]. The LCD can mediate the liquid–liquid phase separation (LLPS) and therefore the formation of stress granules (SGs), which are cytoplasmic condensates lacking a membrane, composed primarily of RNA and RNA-binding proteins (RBPs), generated under unfavorable environmental conditions. SGs may serve to protect RNA from degradation by inhibiting the initiation of mRNA translation at the cellular level and starting the synthesis of cytoprotective proteins. They are intrinsically dynamic and dissolve quickly upon stress removal [35]. TDP-43 mislocalization and aggregation are observed in approximately 97% of all ALS cases, including all sALS cases, whereas SOD1 (2%) and FUS (1%) inclusions are associated with the remaining cases [36]. Not only are ubiquitin-immunoreactive inclusions most frequently reported in all forms of ALS, but the aggregates may also be reactive to p62, a protein that participates in sequestosome formation and autophagy [37].

To prevent the formation of protein aggregates, the cells are equipped with protein quality control systems, and the presence of chaperone proteins ensures that proteins in their native form fold correctly. These can intervene to avoid misfolding and therefore restore the proteins to the correct conformation shape: the UPS or the ALP. Dysfunction of these systems causes the formation of protein aggregates [38]. In this context, genetic screening has identified disease-associated mutations in many of the proteins identified within ALS inclusions [5]. These findings imply that aggregates are not merely a disease marker but are also strongly linked to the etiology and pathomechanisms of neurodegeneration.

### 3.1. SOD1

**Figure 2:**
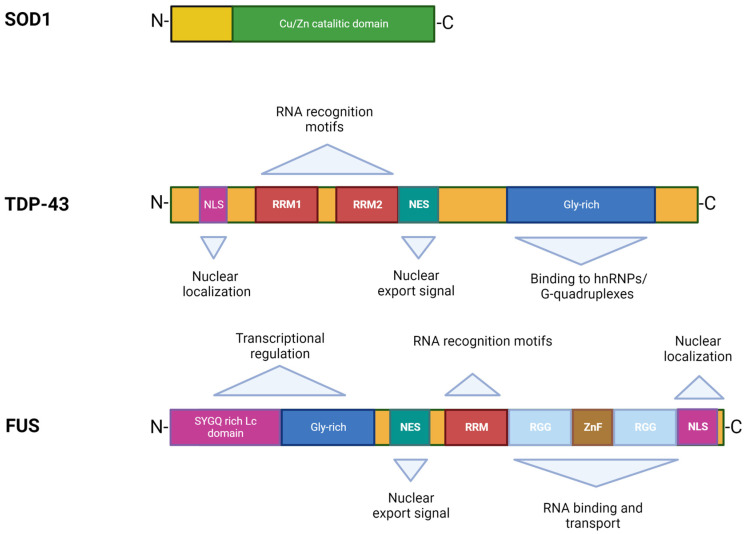
Structures and functional domains of SOD1, TDP-43, and FUS proteins.

In 1993, Rosen et al. first described eleven disease-associated mutations in the *SOD1* gene located on chromosome 21 [39], which encodes for the Cu/Zn superoxide dismutase, a cytoplasmic enzyme responsible for the catabolism of superoxide radicals to hydrogen peroxide and molecular oxygen [40]. SOD1 is ubiquitously expressed, highly conserved, and represents ~1% of all cytoplasmic proteins. It is a 32 kDa polypeptide of 153 amino acids, composed of a binding site with a zinc atom and another for a copper atom (Figure 2). This protein is predominantly localized in the cytoplasm of cells, although it is also apparently present in the intramembrane space of the mitochondria, nucleus, lysosomes, and peroxisomes [41]. Its role is to convert superoxide anions, which are toxic to the cell, into peroxides of hydrogen and oxygen [42]. SOD1 also exerts pro-oxidant activity including peroxidation with the production of hydroxyl radicals and nitration of tyrosines [43]. The ubiquitinated form of this protein is widely expressed and constitutes about 0.5–0.8% of soluble proteins in the human brain [44]. To date, over 200 mutations in *SOD1* have been identified accounting for approximately 20% of fALS patients [45,46], characterized by inter-family and intra-family variability in phenotype with respect to severity of symptoms, age of onset, and disease duration [46]. The most common mutations are G93A, A4V, H46R, D90A, inherited as dominant traits, except for the latter that also shows a recessive inheritance pattern of transmission, but only in Scandinavian populations [32,47].

The two principal critical features of SOD1 mediated cytotoxicity are misfolding and protein aggregation. This means that disease onset is driven by mutant protein that is synthesized inside MNs. MutSOD1 protein interacts specifically with neurofilament light chain mRNA and the dynein–dynactin complex, thus inducing cytoskeletal defects or altering axonal transport [47]. Furthermore, it has an increased tendency to form aggregate-prone monomers, and the degree of instability correlates inversely with survival time, suggesting that increased propensity to aggregation may be the unifying common denominator for different *SOD1* mutations [47]. Researchers identified misfolded SOD1 in MNs in a subset of patients with sALS without *SOD1* mutations, thus suggesting a role for wt SOD1 in sALS, possibly after secondary (oxidative) modification [48,49]. Finally, the aggregation and spread of mutant SOD1 has been demonstrated in cultured cells [50], and its seeding ability via a prion-like mechanism illustrated using spinal cord homogenate [51].

There are currently no explanations for how mutSOD1 causes disease. Initially it was hypothesized that the mutations impair the enzymatic activity of the protein, causing increased levels of reactive oxygen species (ROS) with consequent oxidative stress and death of neuronal cells [52]. More recent studies have shown that mutant protein forms maintain their catalytic activity intact with no apparent causal relationship between residual enzyme activity, clinical progression, and disease phenotype [53].

The mutSOD1 protein accumulates in the oligomeric form and produces cytoplasmic aggregates, which can cause neuronal cell death by sequestering other cytoplasmic proteins required for neuronal survival, blocking the UPS with consequent loss of chaperone proteins, destruction of mitochondria, and the blockade of cytoskeletal or axonal transport [54].

### 3.2. TDP-43

TDP-43 was first isolated in 1995, when researchers observed its ability to bind the transactivation response region (TAR) of HIV DNA, hence the name TAR DNA binding protein [55]. It was subsequently found in the human brain and in several cell culture systems [56]. TDP-43 is a highly conserved protein across different species and shows ubiquitous expression in humans and rodents with a predominant localization in the nucleus. This protein consists of 414 amino acids and has a molecular weight of 43 kDa, encoded by the *TARDBP* gene located on chromosome 1, a member of a heterogeneous family of proteins that bind RNA, known as hnRNP (heterogeneous ribonucleoprotein) [57]. From a structural point of view, the protein contains an N-terminal region, a nuclear localization signal (NLS), two RNA recognition motifs (RRM1 and RRM2) which exhibit an export signal nuclear (NES), and a C-terminal region comprising a glycine-rich LCD which mediates the interaction with other proteins belonging to the hnRNP family (Figure 2) [57]. Mutations affecting the TDP-43 protein represent about 5% of sALS and about 3% of fALS cases [58] as well as patients affected by frontotemporal dementia (FTD) [13,14]. The majority of *TARDBP* mutations are clustered mainly within the glycine-rich C terminal, and have a crucial role in nucleocytoplasmic shuttling, aggregation propensity, and protein–protein interaction [59]. The cellular function of TDP-43 remains unknown, but different studies have shown that this protein plays a fundamental role in a number of biological activities, including regulation of gene transcription, control of splicing processes, and maintaining the stability of mRNA [60]. Protein levels are strictly controlled through a self-regulation system, with particular involvement of the C-terminal region, further supporting the suggestion that mutations in this domain can interfere with the homeostatic control process by altering the recruitment of complexes required for self-regulation [61].

In pathological conditions, this protein tends to form ubiquitinated inclusions in the central nervous system (CNS), in particular the hippocampus, neocortex, and spinal cord. A further feature that distinguishes it from the wt form is its localization: insoluble protein aggregates tend to form in the cytoplasm in the neurons of patients with either pathology, resulting in a decrease of TDP-43 protein in the nucleus, in contrast to healthy subjects where it is expressed in the nucleus [13]. In addition to ubiquitination, hyperphosphorylation of TDP-43 is important for protein aggregation in ALS pathogenesis. In particular, in samples from ALS patients TDP-43 appears to be hyperphosphorylated at the C-terminal level, thus favoring its aggregation [62]. According to the findings of Braak and colleagues, pTDP-43 is widespread throughout the CNS, particularly in the agranular neocortex, and in spinal and bulbar MNs, where the presence of pTDP-43 makes it impossible to distinguish between the two types of MNs [63]. Furthermore, hyperphosphorylation of TDP-43 has been clearly linked to cell death in areas of the CNS, in relation to disease progression [64]. Another study also demonstrated that pTDP-43 aggregates led to the death of dopaminergic neurons in the substantia nigra [65].

Regardless of the presence of genetic mutations, the aberrant localization of TDP-43 in the cytoplasm of neurons in ALS patients appears to be linked to a pathogenetic mechanism associated with a loss of function in nuclear protein responsible for the regulation of mRNA transcription and splicing processes [60]. The formation of cellular inclusions of TDP-43 induces toxicity in the cell, and in this case the protein acquires a toxic function (gain of function) [66,67,68].

Furthermore, the accumulation of TDP-43 within ubiquitinated inclusions (UBIs) can lead to the altered regulation of genes or factors involved in the degradation processes of intracellular proteins, thus contributing to TDP-43 proteinopathy.

TDP-43 levels are strictly regulated by an intrinsic autoregulatory pathway, as demonstrated by observations that inactivation of one copy of the gene does not reduce protein mRNA levels in mice. Autoregulation is believed to be mediated by TDP-43 dependent splicing of an intron in the 3′ UTR region of its own mRNA, and splicing of this gene leads to unstable RNA which undergoes decay [69]. Furthermore, overexpression of wt human TDP-43 in neurons results in neurodegeneration accompanied by decreased locomotor activity, motor impairment, shorter lifespan, and MN loss [70].

TDP-43 is primarily cleaved by caspase 3 into two extremely aggregation-prone fragments with molecular weights of 25 kDa and 35 kDa, namely TDP-25 and TDP-35, respectively [71]. These fragments derive from the entire wt TDP-43 protein chain, induce greater toxicity, and contribute to the loss of function of TDP-43. Therefore, they must be efficiently cleared from cells to prevent their aggregation and the sequestration of other important neuronal components. The clearance of aberrant or misfolded proteins is mediated by the protein quality control system (PQC) [72]. This system is composed of chaperone and co-chaperone proteins that recognize and bind damaged proteins and direct them towards degradation processes.

As mentioned previously, the LCD sequence of TDP-43 controls its ability to undergo LLPS [73], leading to the formation of insoluble aggregates [74]. In particular, when aggregate-prone TDP-43 is more concentrated, the critical concentration for LLPS is exceeded, making it difficult to maintain protein homeostasis by preventing aggregation [62]. Further evidence of the importance of proper interactions between TDP-43 and LLPS comes from a recent study by Gao et al. performed on LLPS-deficient TDP-43 mice, showing that low levels of TDP-43 and LLPS led to the alteration of neuronal cells [75].

### 3.3. FUS

The discovery of TDP-43 mutations in ALS rapidly led to the identification of mutations in another RNA binding protein, namely FUS [16,17], accounting for 4–5% of fALS and 1% of sALS forms associated with young age at onset and short survival time [5,76]. The *FUS* gene is located on chromosome 16 and encodes for a 526-amino acid protein of 75 kDa in weight, showing a structure similar to that of TDP-43 (Figure 2) [77,78]. Although FUS was initially identified as a component of a fusion oncogene resulting from a chromosomal translocation observed in liposarcomas [60], this protein also plays a role in RNA processes [79]. FUS is widely expressed in most human tissues [80] and is primarily localized at the nucleus, although it shuttles to the cytoplasm to mediate a wide range of cellular processes including DNA repair, genomic stability, transcriptional regulation, splicing, transport, and maturation of mRNAs [60,81]. Specifically in the CNS, FUS regulates mRNA transport towards the dendrites and supports synaptic plasticity upon activation of glutamate receptors [82].

As with *TARDBP*, the majority of pathogenic mutations in *FUS* map to the C terminal within the NLS region of the protein that regulates interaction with transportin-1 [83], thereby interfering with the nuclear–cytoplasmic balance of FUS [78,82]. Accordingly, this protein exhibits mainly nuclear localization under healthy conditions, but abnormal cytoplasmic aggregates have been found in the brains and spinal cords of ALS patients with *FUS* mutations [16,57,82]. Therefore, the toxic effects of FUS seem to be related to its aberrant cytoplasmic localization that possibly disrupts nucleocytoplasmic transport [84]. This evidence is supported by a study in *Drosophila* where deletion of the nuclear export signal reduced the toxicity of mutant FUS [85].

Furthermore, authors have reported that FUS can undergo phase transition by LCD sequence [86] and formation of GSs [80], similar to TDP-43 [75]. Study in vitro demonstrated that ALS mutations may accelerate the kinetics of phase separation and exacerbate the transition of FUS from liquid to solid phase [86]. Further studies confirmed that an aberrant phase transition is reflected in the molecular mechanism underpinning ALS pathogenesis [87,88,89,90].

Interestingly, neuropathological examination of tissues from patients harboring *FUS* mutations showed increased cytoplasmic FUS staining, FUS-immunoreactive dystrophic neurites, and cytoplasmic inclusions in lower MNs [16,17]. These mislocalized immunoreactive FUS inclusions were strikingly non-reactive for TDP-43, suggesting that neurodegenerative processes driven by FUS are independent from TDP-43 mislocalization.

## 4. Molecular Mechanisms Leading to Protein Aggregation in ALS

Protein folding is the process by which a protein develops a well-defined three-dimensional structure, i.e., the tertiary structure, the result of a series of polypeptide chain folding [91]. The misfolding of a protein is the basis of the phenomenon of protein aggregation. This occurs mainly due to hydrophobic forces that cause two proteins, similar or different, to interact and form oligomers or amorphous fibrils.

FUS and TDP-43 contain domains enriched for asparagine, glutamine, tyrosine, and glycine residues. Each can adopt one of two conformational states: an explained or unfolded state, and an aggregated state. Prion proteins in an aggregated state can sequester prion proteins in an unfolded state to adopt aggregation-prone conformation, and aggregation thus spreads. It has been shown that the aggregation of SOD1, FUS, and TDP-43 is based in regions similar to prion domains [51,92,93,94]. Many of the mutant proteins in ALS participate in the formation of RNA granules, which is normally a reversible process but under pathological conditions it is assumed that the formation of RNA SGs results in insoluble aggregates. Studies have verified the colocalization of positive inclusions of TDP-43 by observing markers of these SGs in MNs of sALS patients [95,96]. ALS-associated mutant proteins alter the formation of RNA SGs, interfering with local RNA translation and making proteins more easily aggregated. Defects in both the assembly and disassembly of SGs have been associated with neurodegenerative disorders [97]. The size of ALS-associated proteins and their interactors within cytoplasmic aggregates may result in loss of function. Meanwhile, the knockdown of FUS or TDP-43 results in the loss of nuclear foci known as GEM [98,99]. The majority of ALS-causing mutations harbor the LCD regions of SG-related RBPs, resulting in abnormal LLPS abilities that impair SG homeostasis and lead to irreversible and toxic aggregates [100]. In addition, reduced numbers of GEM have been observed in the spinal cords and fibroblasts of patients with ALS [99,101]. These findings indicate the ability of protein aggregates to sequester RNA and the resultant effects on cells.

Protein degradation occurs through the processes of UPS and autophagy, which are crucial for the removal of ubiquitinating proteins and are significantly implicated in the presence of protein aggregates. As mentioned above, chaperone molecules are involved in the folding of proteins and preventing protein aggregation in response to stress under physiological conditions, and also help in protein degradation in the proteasome and during the process of autophagy. Chaperones are overregulated in ALS and are present in MS aggregates [102], leading to increased solubility and reduced toxicity of FUS and TDP-43 [103,104]. Accordingly, the knockdown of molecular chaperones increases the accumulation of TDP-43 C-terminal fragments and increases the toxicity of TDP-43 overexpression [105], while proteasome inhibition was found to increase levels of endogenous TDP-43 and associated toxicity [106]. The formation of intracellular aggregates may depend on the accumulation of misfolded and generated proteins, or could be a direct consequence of the mutation or of oxidative stress. In both cases, malfunctioning of the misfolded protein response system (UPR) appears to play a key role [102].

The transport of misfolded proteins towards particular compartments, favoring their destruction, is a critical aspect of preventing protein accumulation and aggregation [107]. The mechanism behind the sequestration of ALS-related proteins (FUS, TDP-43, and SOD1) prevents interactions that could result in cytotoxic oligomers and cell damage [107,108]. The three types of cellular compartment include juxtanuclear quality control (JUNQ), insoluble protein depots (IPOD), and RNA-specific interaction compartments/inclusions (RISCI) [107,109]. Due to their colocalization with ubiquitin, JUNQs are dynamic and their ubiquitination process occurs more rapidly [107,109]. In contrast, IPOD inclusions are not dynamic and involve a longer and slower ubiquitination process [107]. Published research has reported that the sequestration of mutSOD1 proteins in JUNQ may explain why this protein becomes toxic. Indeed, it appears that mutSOD1 protein can impair the dynamic nature of the JUNQ compartment [108,110]. Furthermore, several studies have demonstrated that SOD1 aggregations might affect the UPS system and damage the cellular signaling that is essential for homeostasis [111,112]. When co-expressed, it appears that TDP-43 aggregates, which were initially believed to be located in IPOD, are localized differently within SOD1-positive inclusions (JUNQ) [109].

Numerous research efforts continue to be focused on the toxic effects of protein aggregates on ALS patients. Explanations have been proposed for how ALS protein inclusions result in extreme cytotoxicity. It has been revealed that in cultured cell models these inclusions have the ability to sequester other proteins necessary for their function, such as those involved in the proper development of the cytoskeleton, chromatin architecture, and RNA metabolism [113,114,115]. In an intriguing study, Woerner et al. found that protein accumulation in the cytoplasm inhibits nucleocytoplasmic transport, including mRNA transport, and as a result blocks the annexes of the cellular pathways [116].

Furthermore, significant mitochondrial degradation and dysfunction have frequently been linked to TDP-43, SOD1, and FUS. The apparent cause of this is the blockage of mitochondrial transport pores and the subsequent accumulation in the intermembrane space, thereby activating the mitochondrial UPR [117,118,119]. The sequestration of additional molecules and cellular elements necessary for cellular homeostasis may be another mechanism through which these aggregates instigate neuronal death. Together these phenomena result in loss of function in essential proteins [120]. In conclusion, several studies have demonstrated that protein aggregation causes complete disruption of primary protein-degradation pathways, from the UPS system to changes in the autophagy process [121,122]. However, future studies are necessary to investigate how these processes are affected in ALS, as they may represent potential therapeutic targets.

## 5. TDP-43, SOD1 and FUS Aggregates in Other Neurodegenerative Diseases

**Figure 3:**
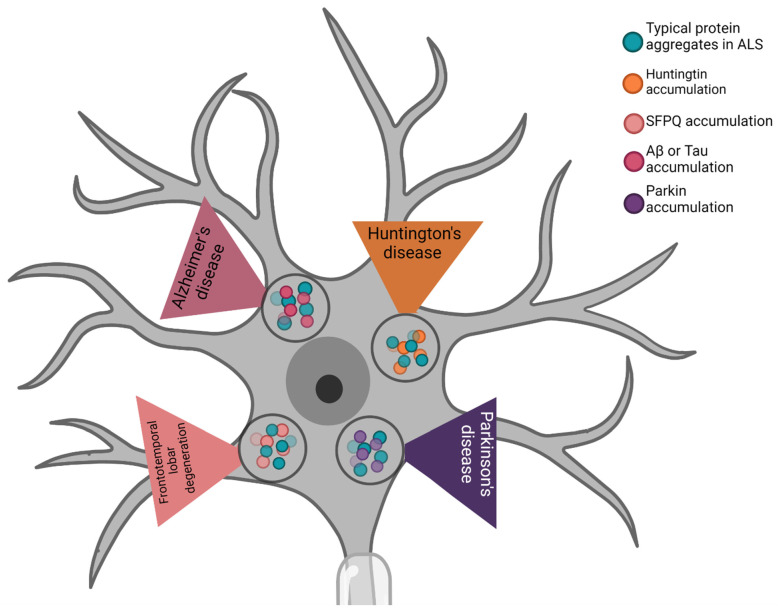
The protein aggregates typically associated with ALS are linked to protein inclusions in other neurodegenerative diseases, also promoting neuronal death in these pathologies.

These altered proteins typically associated with ALS have also been found in other neurodegenerative disorders. In this section of our study we briefly review their involvement in frontotemporal lobar degeneration (FTLD), AD, PD, and HD (Figure 3).

In addition to ALS, FUS and TDP-43 are also associated with FTLD [123,124]. The TDP-43 aggregates in FTD are characterized by compact neuronal cytoplasmic inclusions (NCIs) and short dystrophic neurites (DNs) with neuronal intranuclear inclusions (NIIs), which are preferentially localized in the upper neocortical layers. These characteristics are particularly associated with the behavioral variant of FTD (bvFTD). Other types of TDP-43 aggregates are linked to FTD without the presence of MN disease (MND), and to semantic frontotemporal dementia (SD). These data provide evidence for the critical role of TDP-43 in FTLD pathology [124,125]. A subset of FTLD with ubiquitinated inclusions has been identified in the presence of FUS protein accumulations. A study published in 2009 showed that FUS aggregates formed only in the affected areas of the cortex in FTLD brains. It has also been demonstrated that FUS plays a significant role in neuronal homeostasis, and that the multifunctional interaction between FUS and SFPQ is a harmful factor in FTLD diseases [126,127].

Accumulations of these proteins have also been found in PD and AD. PD affects the dopaminergic neurons in the substantia nigra, and mutant TDP-43 has been found in PD patients [128]. Cytoplasmic aggregates were found in the CNS of PD patients, in particular the spinal cord and bulbar nuclei. TDP-43 aggregates in PD are reportedly associated with induced dopaminergic neuronal loss in PD patients with altered Parkin expression. In some physiological conditions, Parkin over-expression can alleviate neuronal death induced by TDP-43 [129,130].

In recent years, TDP-43 accumulation has been reported in several AD cases [131,132,133]. Studies have shown that these cytoplasmic aggregates have significative negative effects in patients, including brain atrophy and memory loss [134,135], and these effects are due to direct interaction between TDP-43 and Aβ or tau [133,136]. Furthermore, it has been suggested that TDP-43 accumulation may aggravate AD pathology. Moreover, various studies of these two diseases have suggested a role for SOD1 protein in neuronal death in the substantia nigra of patients with AD or PD. As occurs in ALS, the protein aggregation of SOD1 along with the alteration of proteins typically involved in AD and PD causes increased damage following oxidative stress, in turn leading to neuronal death [137,138]. In PD, SOD1 aggregation is localized only in the regions with neuronal loss. A recent study reported clear evidence of the role of this protein in other degenerative neurological pathologies [137], while previous studies revealed that SOD1 promotes the formation in AD brains of protein aggregates associated with Aβ plaques and neurofibrillary tangles [139].

The final neurodegenerative disease briefly covered by this review is HD, a disorder caused by huntingtin (HTT) inclusions in the CNS (Table 1). TDP-43 accumulation has been found in this disease, but the question whether this protein co-localizes with HTT to form inclusions remains controversial [140]. Sampedro and colleagues recently demonstrated that alterations of TDP-43 in the plasma of HD patients were correlated with typical features of this pathology and played a role in the severity of typical HD symptoms [141]. A very recent study revealed that cells responded in different manners to various types of aggregate: the presence of HTT-polyQ aggregation induced a proteotoxic stress response, while aggregation of mutant FUS led to malfunctioning proteostasis in the HEK293T human cell line and primary neuronal cells [142]. In these contexts, the same authors further considered the possible role of molecular chaperones, which are important regulators of protein folding and pathological aggregation. They found that cells obtain protection from mutant FUS aggregation by a complex of full-length (FL) DNAJB14 and DNAJB12 interacting with HSP70 [142]. However, DNAJB12-FL exacerbated HTT-polyQ aggregation, suggesting differential roles for DNAJ isoforms in the regulation of different aggregated proteins [142].

## 6. Misfolding Proteins Typical of Other Neurodegenerative Diseases in ALS

In addition to recognized ALS hallmark proteins, recent research has uncovered novel proteins that appear to be involved in the disease. In the CNS cells of ALS patients, aggregations of additional proteins such as α-syn, tau, or Aβ have been identified, suggesting their involvement and complex interplay in the pathophysiology of ALS [143].

Recent research by Calvo et al. has shown that ALS exhibits characteristics resembling those of other pathologies, particularly synucleinopathies [144]. Several studies have reported the presence of α-syn aggregates in the spinal cords and glial cells of ALS patients, and these apparently play an important role in neuronal degeneration and related onset of symptoms typically associated with PD [145,146]. Additionally, evidence of interaction between a-syn aggregates and SOD1 has been found in numerous investigations, specifically in mutant transgenic mice hSOD1^G93A^ and in the brain tissue of ALS patients [24,147,148].

Recent medical research has shown that tau may serve as a biomarker for ALS diagnosis. In fact, the study of cerebrospinal fluid (CSF) from ALS patients revealed significantly altered levels of tau and associated protein accumulations [149,150]. Furthermore, a fascinating study published in 2016 identified that neurotoxic tau fragments were detected in brain and spinal cord samples from sALS patients, but not in healthy individuals [151]. A polyclonal antibody that recognizes pThr 175 tau was used in the analysis of tau-immunoreactive inclusions in ALS patients, revealing that widespread alteration of tau is also associated with an increase in TDP-43 immunoreactivity [23].

The literature also provides evidence of a potential role for Aβ accumulation during the process of neuronal neurodegeneration occurring in ALS [152]. In fact, it has been discovered that Aβ accumulates in the anterior horn of the spinal cord at the MN level in patients with ALS. Furthermore, the aberrant accumulation of Aβ42 in spinal cord MNs of ALS patients is linked to oxidative stress-induced cytotoxicity and may contribute to neurodegeneration [153]. Clinical observations resulting from the analysis of Aβ levels in CSF suggested that the tau protein may be a potential biomarker for the diagnosis of ALS [154].

## 7. Conclusions

To date, it is not yet completely understood whether and how these protein aggregates cause cell death in ALS, although studies on TDP-43, SOD1 and FUS aggregates have been illuminating. There are a number of possible mechanisms for how protein aggregation in ALS might result in cell toxicity, and there is strong evidence linking many aggregating proteins, including SOD1, TDP-43, and FUS, to mitochondrial dysfunction and indeed complete mitochondrial degeneration [117,155]. Although the exact mechanism is yet to be elucidated, the dysfunction may be caused by the blockage of mitochondrial transport pores, accumulation inside the intermembrane space, and/or activation of the mitochondrial unfolded protein response. It is also recognized that protein aggregation interferes with the proteostasis network, affecting the key protein degradation pathways of autophagy and the UPS [121]. The sequestration of other vital cellular molecules into protein aggregates is likewise detrimental to cell health by inducing loss of function in essential proteins [120]. It has been shown to affect the expression of amyloidogenic sequesters of other proteins involved in crucial biochemical pathways such as proteostasis, cytoskeletal maintenance, chromatin organization, and RNA metabolism [114], some of which are disrupted in ALS [5,113]. Finally, evidence also suggests that protein aggregation in the cytoplasm prevents mRNA transport as well as other types of nucleocytoplasmic transport, compromising global RNA metabolism. Given the variety of downstream consequences that protein aggregation has on cellular health, it remains difficult to identify specific interactions that would be viable therapeutic targets, aside from the main aggregating proteins themselves [116].

The identification of biomarkers and therapies for early diagnosis and effective treatment of ALS will be aided in future by a better understanding of the precise role of protein aggregation and changes of proteostasis within the pathological mechanisms of the disease.

## Figures and Tables

**Table 1:** ALS proteins also found in other neurological diseases.

Neurodegenerative Disease	ALS Proteins	Specific Protein
Alzheimer’s disease	TDP-43	Aβ
	SOD1	Tau
Frontotemporal lobar degeneration	FUS	SFPQ
	TDP-43	
Huntington’s disease	FUS	HTT
	TDP-43	
Parkinson’s disease	TDP-43	Parkin
	SOD1	

## Data Availability

Not applicable.
